# Is body composition important in the context of renal function in pediatric neurogenic bladder?

**DOI:** 10.1007/s00467-024-06557-5

**Published:** 2024-10-17

**Authors:** Joanna Bagińska-Chyży, Adrianna Błahuszewska, Agata Korzeniecka-Kozerska

**Affiliations:** 1 Department of Pediatrics and Nephrology, Medical University of Białystok, 17 Waszyngtona Str, 15-274 Białystok, Poland; 2 Department of Rheumatology and Internal Medicine, Medical University of Bialystok, 24A Skłodowska-Curie Str, 15-276 Białystok, Poland

**Keywords:** Glomerular filtration rate, Cystatin C, Bioimpedance, Neurogenic bladder, Myelomeningocele

## Abstract

**Background:**

Neurogenic bladder due to myelomeningocele (MMC) is a significant risk factor for chronic kidney disease in children. Cystatin C (CysC) is a more accurate GFR marker than creatinine as it is unaffected by muscle mass but may be influenced by fat mass and BMI. This study evaluates: (1) GFR measurement accuracy using CysC and creatinine in MMC-related neurogenic bladder, (2) the relationship between body composition via bioelectrical impedance analysis (BIA) and renal parameters, and (3) the use of BIA for non-invasive GFR and body composition assessment.

**Methods:**

Forty children (median age 9.96 years) underwent serum creatinine, CysC testing, and BIA measurements. We assessed age, sex, spinal lesion level, anthropometric measurements, BMI, and activity using Hoffer’s scale. GFR was calculated using five creatinine-based formulas, three CysC-based, and three combining CysC and creatinine, including BIA GFR as an alternative approach.

**Results:**

Creatinine-based GFR estimates were significantly higher than CysC-based GFR. Although only 30% of MMC patients met the traditional BMI criteria for overweight/obesity, 62.5% were obese based on BIA-measured body fat percentage. Significant differences were found in CysC and CysC-based GFR equations within BMI and fat mass groups. Positive correlations were observed between CysC and body weight, BMI percentiles, body fat mass and fat-to-muscle ratio. Muscle mass positively correlated with creatinine.

**Conclusions:**

BIA-determined fat mass percentage is a more sensitive obesity indicator than BMI in MMC patients. CysC levels and CysC-based GFR equations are influenced by body fat mass, requiring consideration of adiposity to avoid misestimating renal impairment.

**Graphical abstract:**

**Figure:**
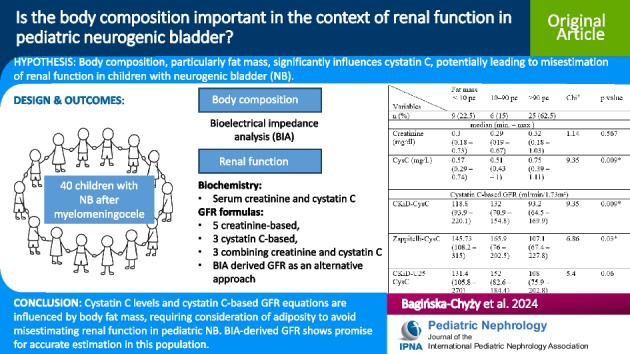
A higher resolution version of the graphical abstract is available as supplementary information

**Supplementary Information:**

The online version contains supplementary material available at 10.1007/s00467-024-06557-5.

## Introduction

Myelomeningocele (MMC), a neural tube defect with an estimated prevalence of 3.9 per 10,000 births, considering average value of the last 20 years in Poland, presents multiple complications in pediatric patients, impacting various organs and systems [1, 2]. Some of the key complications encompass neurological problems and orthopedic issues like musculoskeletal deformities and mobility difficulties including varying degrees of lower limb paralysis that impede walking, often necessitating assistive devices or wheelchairs. MMC is also the leading cause of pediatric neurogenic bladder (NB). Dysfunctional bladder control leads to recurrent urinary tract infections, urinary incontinence, and potential kidney damage [3]. Prolonged urinary tract problems can lead to chronic kidney disease, which is over three times more prevalent than in the general population, requiring regular monitoring and intervention [4].

The glomerular filtration rate (GFR) is regarded as the best overall parameter for evaluating excretory kidney function in clinical practice. Several methods are available to assess GFR in pediatrics, but obtaining accurate and precise measurements of GFR in MMC patients remains challenging. Creatinine-based equations, such as the Schwartz, updated Schwartz, or Counahan–Barratt formulas, rely on serum creatinine, which is primarily influenced by muscle mass and requires the child's height. The precision and accuracy of these methods is limited in MMC patients due to decreased overall muscle mass resulting from muscle atrophy caused by denervation and inactivity [5]. Height measurements are further complicated by orthopedic issues, including kyphosis, scoliosis, abnormal vertebrae, and lower extremity hypoplasia associated with their condition.

Based on scientific literature, cystatin C (CysC) has proven to be a superior indicator of GFR compared to serum creatinine [5, 6]. It acts as a cysteine protease inhibitor, has a low molecular weight and is produced by all nucleated cells at a constant rate, including adipocytes. The most commonly used CysC equation internally validated for MMC patients was developed by Zappitelli et al. [7]. Estimated GFR derived from CysC is often considered more accurate for assessing GFR in the context of sarcopenia, as it is not influenced by muscle mass compared to creatinine. However, some studies have reported that increased adiposity or obesity associates with higher circulating concentrations of CysC [8, 9]. Children and adolescents with MMC typically have greater total body fat compared to their healthy peers, primarily due to excess adiposity in the lower extremities. This is unsurprising given that MMC predominantly impacts the lower extremity muscles, leading to increased subcutaneous and intramuscular fat in areas with chronic muscle paresis. Bioelectrical impedance analysis (BIA) offers an accurate and straightforward method for assessing body composition parameters.

In our study, we evaluated (1) the accuracy of GFR measured using CysC and creatinine in children with NB due to MMC, (2) the association of muscle mass and fat mass obtained by BIA with renal parameters, and (3) the applicability of noninvasive BIA as a tool to estimate GFR and body composition parameters.

## Methods

### Patients

This prospective analysis was conducted on 40 children. The inclusion criteria were as follows: 1) Patients aged 1–18 years with NB after MMC, 2) Children who underwent BIA with complete anthropometric parameters, serum creatinine and CysC measurements. Exclusion criteria: 1) Patients with NB from different etiology than MMC. The lesion level in MMC patients was reported intraoperatively and radiologically and scored from 1 to 3 (1–thoracolumbar, 2–lumbosacral, 3–sacral lesion). The ambulatory function of MMC patients was classified using Hoffer’s scale into non-ambulators, non-functional ambulators, household walkers, and community walkers [10]. Non-functional ambulators, who walk only during therapy, were grouped with non-ambulators, who are entirely wheelchair-dependent, due to their similar main mode of ambulation in daily life.

### Body composition parameters

BIA measurements were performed using the BioScan 916S device (Maltron International, Essex, United Kingdom) according to the manufacturer’s instructions. This device delivers a current of 0.7 mA with an impedance of 100 to 1000 Ω, precision of ± 3 Ω, and a frequency of 50 kHz. The measurement was applied on the right side of the body, with the participant lying supine. Among numerous body composition measures provided by BIA, we focused our attention on the values of muscle mass (kg and %), fat mass (kg and %) and body mass index (BMI, kg/m^2^) that were calculated from BIA data, resistance, and reactance. We reported patients as overweight and obese using the WHO growth reference standards for BMI z score (z-BMI) and percentiles [11]. The WHO advises that children with BMIs below the 5th percentile are classified as underweight. Those with BMIs around the 85th percentile (between 1 and 2 SDs above the mean; z-BMI > 1 and ≤ 2) are considered overweight, while children at the 97th percentile (more than 2 SDs above the mean; z-BMI > 2) are categorized as obese. Additionally, we calculated the fat-to-muscle ratio (FMR) as body fat mass (kg) divided by muscle mass (kg). BIA-derived body fat percentage was applied to body fat percentile reference curves for children [12]. According to the authors’ recommendation, we use the 90th and 97th sex-specific body fat percentiles to identify overweight and obesity. Muscle mass percentage was applied to the reference curves for children by gender and exact age by McCarthy et al. [13]. The values at the lowest centiles (≤ 2nd centile, ≤ 9th centile) were used to define sarcopenia. The reference curves for body fat and muscle mass percentiles in pediatric populations are restricted and may not comprehensively cover all age groups. Specifically, the available percentiles for fat mass percentage are applicable to children aged 3 to 16 years, whereas those for muscle mass pertain to children aged 5 to 19 years. Due to these age limitations, the reference curves did not encompass the entire study sample, resulting in the absence of fat mass percentiles for 3 children and muscle mass percentages for 5 children within the cohort.

### GFR measurements

The equations used to determine eGFRs are shown in Table 1. The creatinine-based GFR was calculated with 3 formulas: the original Schwartz, updated Schwartz, Counahan–Barratt. Additionally, we obtained BIA GFR provided by the BioScan 916 according to manufacturer’s formula incorporating serum creatinine. The CysC-based GFR was calculated with 2 formulas. An additional 2 combined GFR equations were used, including CKiD-Cr-CysC and the Zappitelli formula, that incorporates creatinine and CysC and has a modifier term for MMC patients. Recently, Chronic Kidney Disease in Children Under 25 Study (CKiD-U25) equation was published and 3 formulas were presented for patients between 1 and 25 years of age incorporating serum creatinine-only, cysC-only, or both [14]. For convenience, we employed a web-based calculator available online to facilitate the clinical and research application of these newly published formulas [15]. All the GFRs were standardized for a body surface area (BSA) of 1.73 m^2^ and expressed in ml/min per 1.73m^2^. BSA was calculated with the Dubois formula: BSA = height^0.725^ × weight^0.425^ × 0.007184.

**Table 1:** Equations used to calculate GFR in ml/min per 1.73 m^2^

Formula	Equation
Cr-based only	
The Original Schwartz	GFR = k × L (cm)/Cr (mg/dL), where k ~ 0.33 (preterm infant), k ~ 0.45 (full term to 1 year of age), k ~ 0.55 (children to 13 years of age and adolescence females), k ~ 0.7 in adolescent males after age 13 years
The Updated Schwartz	GFR = 0.413 × L (cm)/Cr (mg/dL)
Counahan–Barratt	GFR = 0.43 × L (cm)/Cr (mg/dL)
CysC-based only	
CKiD-CysC (2012)	GFR = 70.69 × (cysC)^−0.931^ (mg/L)
Zappitelli-CysC	GFR = 75.94/(cysC)^1.17^ (mg/L)
Cr-CysC-based	
CKiD-Cr-CysC (2012)	GFR = 39.8 x [L/Cr]^0.456^ × [1.8/cysC]^0.418^ × [30/BUN]^0.079^ × [1.076^male^] [1.00^female^] × [L/1.4]^0.179^
Zapitelli-Cr-CysC	GFR = [43.823 × e^0.0033 x L (cm)^]/[CysC^0.635^] × [Cr^0.547^] × 1.573 × Cr^0.925^

*L*, length; *Cr*, creatinine; *CysC*, cystatin C; *BUN*, blood urea nitrogen

### Biochemistry

Serum creatinine was measured by standard methods from a kinetic colorimetric compensated Jaffe technique (Roche Modular, Meylan, France) on biochemical analyzer Cobas 6000 C501. Serum CysC samples were assessed with the Siemens N–latex CysC kit, the values obtained were recalculated according to the recommendations of the manufacturer. We adjusted the CysC values, applying a correction factor of 1.17 to align them with the equivalent values calibrated by the International Federation of Clinical Chemistry (IFCC) [16].

### Statistics

The data were collected in a Microsoft Excel database. Statistical analysis was performed using Statistica 13.0. (StatSoft Inc, Tulsa, OK, USA). Continuous variables were expressed as the median and range, unless stated otherwise. All studied parameters were analyzed using nonparametric tests: Mann–Whitney, Kruskal–Wallis, Chi^2^ and the sign test. Correlations were assessed with the Spearman test. Values of *p* < 0.05 were considered significant.

This study was approved by the Ethics Committee of the Medical University of Bialystok, which complies with the World Medical Association Declaration of Helsinki regarding ethical conduct of research involving human subjects and/or animals. Patients and their caregivers were enrolled into the study after obtaining informed consent.

## Results

### Study group

The baseline clinical and anthropometric data of the study population are given in Table 2. Among 19/40 (47.5%) girls and 21/40 (52.5%) boys enrolled, the median age was 9.96 (1.58–17.67) years. The studied group was divided according to age: younger and older than 10 years. The majority of patients, 24/40 (60%) had lumbosacral spinal lesion, 10/40 (25%) thoracolumbar and 6/40 (15%) sacral lesion level. Among all the study subjects, 24/40 (60%) were classified as non-ambulators and non-functional ambulators, 8/40 (13%) as household walkers, and 8/40 (13%) as community walkers.

**Table 2:** Characteristics of patients with NB with the comparison according to the age groups

Variables	NB patients *n* = 40	Patients < 10 years old *n* = 20	Patients > 10 years old *n* = 20	*p*
Median (min.–max.)				
Age (years)	9.96 (1.58–17.67)	5.92 (1.58–9.83)	14.37 (10.08–17.67)	< 0.001*
Height (cm)	129 (70–174)	103.5 (70–134)	150 (110–174)	< 0.001*
Weight (kg)	31 (8.5–95)	16.5 (8.5–56)	42 (20.5–95)	< 0.001*
BMI (kg/m^2^)	17.31 (10.41–35.32)	15.73 (10.41–31.19)	20.16 (16.84–35.32) ż	< 0.001*
BMI (centiles)	49.3 (0–100)	36.6 (0–100)	58.4 (9.3–99)	0.07
Z-BMI Z-score	-0.02 (-8–3.48)	-0.34 (-8–3.48)	0.21 (-1.32–2.39)	0.08
BSA (m^2^)	1.06 (0.31–2)	0.68 (0.31–1.39)	1.3 (0.78–2)	< 0.001*
Serum Cr (mg/dl)	0.32 (0.18–1.03)	0.22 (0.18–0.46)	0.39 (0.22–1.03)	< 0.001*
Serum CysC (mg/L)	0.66 (0.29–1.11)	0.58 (0.29–0.8)	0.75 (0.41–1.11)	0.02*
The Original Schwartz	228.7 (91.8–351.4)	230.6 (123.3–351.4)	215.2 (91.8–309.6)	0.32
The Updated Schwartz	159.9 (68.9–276.7)	177.6 (92.5–276.7)	151.8 (68.9–232.5)	0.04*
Counahan-Barratt	166.4 (71.8–288.1)	184.9 (96.3–288.1)	158 (71.8–242)	0.04*
CKiD-CysC (2012)	105.02 (64.5–220.2)	117.4 (86.8–220.2)	93 (64.5–163.5)	0.02*
Zappitelli-CysC	124.4 (67.4–315.4)	143.1 (97.9–315.4)	106.8 (67.3–217)	0.02*
CKiD-Cr-CysC (2012)	120 (81–174)	125.5 (84–174)	111.5 (81–150)	0.13
Zapitelli-Cr-CysC	90.9 (59–151.9)	74.2 (59–131.5)	100.5 (64.7–151.9)	0.01*
CKiD-U25 Cr (2021)	157.2 (76.8–254.5)	157.5 (76.8–254.5)	149.5 (76.8–220)	0.28
CKiD-U25 CysC (2021)	122.8 (75.9–269.8)	132.8 (101.4–269.8)	106.9 (75.9–189.8)	0.04*
CKiD-U25 Cr-CysC(2021)	141.6 (89.1–203.6)	147 (99.6–203.6)	132.9 (89.1–191)	0.07
BIA GFR	139.2 (59.8–308.9)	153.5 (59.8–308.9)	132.5 (65.8–217.6)	0.23
Fat mass (kg)	7.14 (0.69–45.6)	3.17 (0.69–28.5)	10.2 (1.1–45.6)	< 0.001*
Fat mass (%)	22.3 (3.02–50.9)	19.8 (6.9–50.9)	27.9 (3.02–48)	0.02*
Muscle mass (kg)	8.52 (1.88–25.4)	4.64 (1.88–12.2)	13.6 (6.3–25.4)	< 0.001*
Muscle mass (%)	29.2 (21.2–42.05)	28.3 (21.2–34.4)	29.6 (24.1–42.05)	0.14
FMR	0.74 (0.08–2.34)	0.65 (0.24–2.34)	0.92 (0.08–1.98)	0.1

*NB*, neurogenic bladder; *BMI*, body mass index; *Z-BMI*, body mass index Z-score; *BSA*, body surface area; *Cr*, creatinine; *CysC*, cystatin C; *BIA GFR*, glomerular filtration rate derived by bioelectrical impedance analysis; *FMR*, fat-to-muscle ratio

### Body composition measures

Among our patients, 19/40 (51%) had a normal BMI percentile range (between 5–85 percentiles). The median BMI was 17.31 kg/m^2^, which corresponded to 49.3 percentiles and -0.02 z-BMI. Although only 11/40 (30%) of MMC patients met the traditional BMI definition of overweight and obesity, 26/40 (62.5%) of them were obese based on percentage of body fat provided by BIA (Table 3). In the group of children younger than 10 years, 4/20 (20%) were overweight/obese according to BMI, while 10/20 (50%) were classified as such based on fat mass percentage. For those older than 10 years: 7/20 (35%) were overweight/obese by BMI, while 15/20 (75%) were classified as such based on fat mass percentage obtained by BIA. We calculated muscle mass percentage percentiles for children who were categorized as overweight and obese. Among these, 9/24 (37.5%) with a high amount of BIA-derived fat mass were in the lowest muscle mass percentage centiles (≤ 2nd and ≤ 9th centiles), and they had the highest FMR of 1.24.

**Table 3:** Comparison between BMI and fat mass categories

Variables	Fat mass < 10 pc 10–90 pc	> 90 pc	Chi^2^	*p* value	BMI < 5 pc	5–85 pc	> 85 pc	Chi^2^	*p*value	
*n*(%)	9 (22.5)	6 (15)	25 (62.5)			7 (19)	19 (51)	11 (30)		
median (min.–max.)										
BMI (percentiles)	36.5 (0–98.8)	41 (0–56.4)	60.9 (0–100)	4.06	0.13	0 (0–5)	48.6 (9.3–69.7)	94.6 (85–100)	19.3	< 0.001*
Z-BMI	- 0.34 (-8–2.27)	- 0.23 (-3.34–0.16)	0.28 (- 3.6–3.48)	4.06	0.13	-3.34 (-8– -1.65)	-0.04 (-1.32–0.51)	1.6 (1– 3.48)	19.3	< 0.001*
Creatinine (mg/dl)	0.3 (0.18–0.73)	0.29 (019–0.67)	0.32 (0.18–1.03)	1.14	0.567	0.2 (0.18–0.3)	0.35 (0.18–1.03)	0.4 (0.2–0.76)	8.27	0.016*
CysC (mg/L)	0.57 (0.29–0.74)	0.51 (0.43 – 1)	0.75 (0.39–1.11)	9.35	0.009*	0.54 (0.3–0.75)	0.68 (0.41–1)	0.8 (0.54–1.11)	7.89	0.02*
Cr-based GFR (ml/min/1.73 m^2^)										
The Original Schwartz	205.7 (123.2–283.1)	220.8 (181.8–309.6)	242 (91.8–351.4)	1.36	0.51	269 (201.7–302.5)	207 (91.8–351.4)	230.3 (127.6–294.4)	4.95	0.08
The Updated Schwartz	154.4 (81.5–213)	165.8 (107.2–232.5)	167.8 (68.9–276.7)	1.36	0.51	202.4 (151.4–227.1)	154.9 (68.9–263.9)	161.6 (95.8–276.7)	4.95	0.08
Counahan–Barratt	160.8 (84.8–221.8)	172.6 (111.7–242.1)	174.7 (71.8–288.1)	1.36	0.51	210.7 (157.7–236.5)	161.2 (71.8–274.7)	168.3 (99.7–288.1)	4.95	0.08
CKiD-U25 Cr	137.4 (76.8–185.5)	147.8 (122.5–220.1)	157.3 (76.8–254.5)	1.5	0.45	181.1 (137.4–210)	135.8 (76.8–226.5)	157.1 (89.5–254.5)	2.13	0.344
BIA GFR	136.3 (59.8–201.9)	134.6 (99.7–217.6)	142 (65.8–308.9)	1.05	0.58	159.9 (106.4–308.9)	123.2 (59.8—218.8)	142 (77.2–302.8)	1.82	0.401
CysC-based GFR (ml/min/1.73 m^2^)										
CKiD-CysC	118.8 (93.9–220.1)	132 (70.9–154.8)	93.2 (64.5–169.9)	9.35	0.009*	124.8 (92.6–220.2)	101.9 (70.9–163.5)	87.6 (64.5–125.9)	7.89	0.02*
Zappitelli-CysC	145.73 (108.2–315)	165.9 (76–202.5)	107.1 (67.4–227.8)	6.86	0.03*	154.5 (106.15–315.4)	119.8 (76–217)	99 (67.4–156.2)	7.89	0.02*
CKiD-U25 CysC	131.4 (105.8–270)	152 (82.6–184.4)	108 (75.9–202.8)	5.4	0.06	142.7 (104.4–269.8)	122.5 (82.6–189.8)	104 (75.9–143.8)	8.84	0.01*
Combined GFR (ml/min/1.73 m^2^)										
CKiD-Cr-CysC	127 (84–163)	131.5 (88–150)	115.5 (81–174)	0.83	0.65	137 (122–163)	117 (81–150)	112 (82–174)	7.89	0.02*
Zapitelli-Cr-CysC	92.4 (68.9–131.5)	94.2 (66.9–109.1)	90.7 (59–151)	0.15	0.93	90.7 (60.25–131.5)	93.2 (59–151.9)	89.22 (62.3–112.5)	3.39	0.18
CKiD-U25 Cr-CysC	140.7 (99.6–203.6)	156.1 (102.5–191)	134.9 (89.1–199.1)	1.15	0.567	163.8 (142.7–203.6)	134.9 (94.3–191)	129.2 (89.1–199.1)	9.72	0.007*
BIA parameters										
Fat mass (kg)	1.15 (0.69–3.03)	5.18 (1.77–11.7)	9.76 (2.34–45.6)	14.5	< 0.001*	2.42 (0.88–9.76)	7.95 (1.06–14.24)	24.1 (3.1–46.1)	15.53	< 0.001*
Fat mass (%)	10.3 (3.02–15.24)	20.1 (16.5–23.3)	31.5 (25–50.9)	18.4	< 0.001*	18 (10.3–31.5)	20.7 (3–38.9)	37.6 (15.15–50.9)	12.23	0.002*
Muscle mass (kg)	8.16 (1.77–23.9)	4.33 (2.48–17.2)	9.67 (2.97–25.4)	3.78	0.15	4.33 (2.48–7.88)	9.67 (3.1–23.9)	13.21 (2.97–25.4)	8.27	0.016*
Muscle mass (%)	31.7 (22.1–38.6)	29.2 (27.7–40)	28.2 (21.21–42)	3.78	0.15	29.2 (25.4–34.4)	30.45 (24.9–42.05)	27.07 (21.21–29.6)	7.15	0.03*
FMR	0.64 (0.48–0.94)	0.35 (0.08–0.54)	1.24 (0.26–2.34)	14.5	< 0.001*	0.62 (0.35–1.24)	0.68 (0.08–1.46)	1.35 (0.51–2.34)	12.23	0.002*

*BMI*, body mass index; *Cr*, creatinine; *CysC*, cystatin C; *FMR*, fat-to-muscle ratio

^*^p < 0.05

We examined the associations of body composition and renal parameters with physical activity among MMC patients according to Hoffer’s scale. Statistically significant difference was found in creatinine concentration between abovementioned groups (Chi^2^ = 7.28, *p* = 0.03) whereas CysC concentration did not differ (Chi^2^ = 1.67, *p* = 0.43). Differences were recorded in fat mass and muscle mass but they were not statistically significant. Non-ambulators and non-functional ambulators had the lowest creatinine concentrations and muscle mass provided by BIA and the highest percentage of fat mass and FMR in contrast to children with different stages of walking impairment. More detailed data are shown in Table 4.

**Table 4:** Comparison between Hoffer’s scale groups

Variables	NA	HW	CW	Chi^2^	*p* value
*n* (%)	24 (60)	8 (20)	8 (20)		
Median (min.–max.)					
Body weight (kg)	24.8 (8.5–95)	32 (16.4–75)	31 (11–57)	0.1	0.97
BMI percentiles	49.1 (0–100)	57.3 (5–98.8)	40.6 (0–67.9)	1.76	0.41
BMI Z-score	- 0.02 (-8–3.48)	0.18 (-1.65–2.27)	- 0.24 (-3.34–0.51)	1.76	0.41
Fat mass (kg)	8.17 (0.69–45.6)	8.87 (2.5–27.8)	4.37 (1.06–14.24)	2.88	0.41
Fat mass (%)	24.6 (5.14–50.9)	22.2 (15.1–37.9)	13.7 (3–28.5)	2.76	0.42
Muscle mass (kg)	6.94 (1.88–25.4)	9.55 (4.6–23.9)	10.5 (3.07–23.9)	2.52	0.47
Muscle mass (%)	28.3 (21.1–40)	28.7 (25.4–38.6)	31.5 (27.7–42)	3.52	0.31
FMR	0.93 (0.12–2.34)	0.74 (0.49–1.35)	0.42 (0.08–0.91)	3.52	0.31
Serum creatinine (mg/dl)	0.26 (0.18–0.73)	0.42 (0.21–0.72)	0.35 (0.23–1.03)	7.28	0.03*
CysC (mg/L)	0.69 (0.29–1.11)	0.63 (0.49–1)	0.55 (0.4–0.76)	1.67	0.43

*NA*, non-ambulators and non-functional ambulators; *HW*, household walkers; *CW*, community walkers; *CysC*, cystatin C; *FMR*, fat-to-muscle ratio

^*^p < 0.05

### Renal parameters

#### Correlations of anthropometrics and body composition measures with serum creatinine and CysC

Childhood height, weight, BSA, BMI percentiles and z-BMI were positively correlated with creatinine (r = 0.737, r = 0.722, r = 0.719, r = 0.349, r = 0.357 respectively; *p* value < 0.05) and CysC (r = 0.356, r = 0.416, r = 0.374, r = 0.424, r = 0.422 respectively; *p* value < 0.05). Statistically positive correlations were also found between fat mass (in kg and %) and FMR for CysC (r = 0.485, r = 0.432, r = 0.393 respectively; *p* value < 0.05). Muscle mass, both in kilograms and percentage, showed a statistically significant positive correlation with creatinine (r = 0.767, r = 0.343 respectively; *p* < 0.05). CysC also had a statistically significant positive correlation with muscle mass in kilograms (r = 0.366; *p* < 0.05), but there was no significant correlation between CysC and muscle mass percentage (r = -0.19; *p* < 0.05). Significant differences in CysC concentrations were observed across different BMI and fat mass groups, whereas creatinine levels remained stable (Table 3).

### GFR measurements

Comparison of the GFR measurements is presented in Table 2. GFR values were compared according to the age groups: above and below 10 years old. As shown, the original Schwartz and Zappitelli-Cr-CysC with modification for MMC patients yielded the highest and lowest median GFR values, respectively. This represented a big difference up to 137 ml/min/1.73 m^2^. In older children, kidneys undergo aging-related changes expressed by a higher serum concentration of creatinine, CysC and decline in the GFR.

### GFR estimations based on creatinine levels

The original Schwartz, the updated Schwartz and Counahan–Barratt formulas had the highest median of GFR values. From all 5 formulas based on creatinine level, the median of BIA GFR was the lowest one and stayed in a good agreement with the latest CKiD-U25 Cr-CysC GFR equation. There was no statistically significant difference between these two equations (*p* = 0.645). A significant negative correlation was found between BIA GFR and creatinine (r =—0.686, *p* < 0.05).

### GFR estimations based on CysC levels

The median GFR values were lower than those based on creatinine. Within the fat mass groups and BMI groups, there were statistically significant differences in CysC-based GFRs. Children with the highest percentage of body fat and BMI indicated overweight/obesity had the lowest median GFR-CysC values (Table 3).

## Discussion

Patients with NB due to MMC exhibit a higher risk of progression to chronic kidney disease. Therefore, a comprehensive follow-up of kidney function should be routinely conducted, starting early in the course of the disease, as they are susceptible to developing permanent renal damage. According to the literature, most patients have normal upper urinary tracts at birth; however, nearly 60% will experience upper tract deterioration [17]. Identifying early indicators of renal damage is crucial to preserving kidney function at the earliest possible stage.

To identify any age-specific differences in renal parameters during the course of the disease, a comparison was made between children younger than 10 years and older. It revealed significant differences in creatinine, CysC, and GFR estimations, with the exception of the Zappitelli-Cr-CysC equation. In line with the initial analysis of age-related GFR differences in children with MMC by Szymanski et al. [18], the Zappitelli-Cr-CysC equation was the only pediatric GFR formula that unexpectedly increased with age. Further large-scale studies are required to assess the utility of the Zappitelli equation in patients with MMC. While the authors [18] employed four pediatric formulas, our study extended these calculations by incorporating the latest CKiD U25 formulas and BIA GFR, both of which show a decline with age. The observed increase in creatinine and CysC levels, accompanied by a corresponding decline in GFR in older children, indicates a loss of kidney function that may be attributed to prolonged exposure to risk factors such as recurrent urinary tract infections and elevated detrusor filling pressures [19].

Not only renal parameters, but also body composition evolves over time, as a child with MMC ages, commonly resulting in obesity. BMI is the conventional metric for assessing body composition in the general population; however, evidence indicates that BMI-based obesity diagnoses using height may not be reliable in patients with MMC [20, 21]. BMI is an insufficient measure of total body fat. In our study, a notable increase in the number of subjects classified as overweight and obese was observed when fat mass percentage assessed by BIA was used. Our findings align with those of Liu et al. [22] in an adult cohort of patients with spina bifida, where 43.8% were classified as obese using traditional BMI calculations based on height. When BMI was adjusted using arm span or trunk fat percentage determined by dual-energy X-ray absorptiometry (DXA), a higher obesity rate was observed in the same population. There is limited data on body composition measurements in the pediatric MMC population. Recently, a few studies, including Polfuss et al.'s analysis [21] in children with intellectual and developmental disabilities, including MMC, have emerged. They found that BMI-based classifications underestimated the children's weight status compared to DXA-derived body fat measurements. Unlike the aforementioned analyses, this study investigates the utility of BIA instead of DXA. Although DXA is considered the gold standard for body composition assessment, it is more time-consuming, costly, and less accessible in clinical settings compared to BIA. There is a paucity of data on the concordance between BIA and DXA in measuring body composition in pediatric MMC populations. In other populations, such as patients with chronic kidney disease, the majority of studies [22, 23] indicate that BIA exhibits good agreement with DXA in quantifying fat and lean tissue. Consequently, further research is warranted to comprehensively evaluate the accuracy of BIA in patients with MMC.

In our study, both fat mass and muscle mass, as measured by BIA, increased with age; however, the muscle mass percentage within the overall body composition remained stable. The higher-than-expected body fat relative to BIA observed among our patients may be partially attributed to lower-than-expected muscle mass, a condition known as 'sarcopenic obesity'. This condition, characterized by adipose tissue expansion and muscle loss, leads to increased levels of pro-inflammatory cytokines and is not unique to chronic conditions affecting the nervous system [24, 25]. In our study, 37.5% of overweight/obese children exhibited low muscle mass percentage according to age- and sex-specific percentiles. Therefore, overweight and obesity do not preclude concurrent muscle wasting, which, combined with higher body fat, may negatively impact patients with MMC. The detrimental effects of excessive fat mass on physical functioning are well-documented when considering BMI in children, yet the role of muscle mass in conjunction with fat mass has received less attention. In our study, we employed the FMR, an alternative approach for assessing body fat proposed in the adult population [26, 27]. To our knowledge, this is the first application of FMR in a population of MMC patients. FMR is utilized to evaluate metabolic syndrome in adults, with higher FMR being a risk factor for various conditions, including type 2 diabetes, cardiovascular diseases, and mortality. In our study, non-ambulatory and non-functionally ambulatory patients, as well as obese patients, exhibited the highest FMR rates. This may indicate that these subgroups of MMC patients are at high risk for metabolic syndrome and its long-term consequences. However, further studies are needed to substantiate these findings.

Our main focus was to assess the influence of body composition measures on renal parameters. A number of determinants of both serum creatinine and CysC, including BMI and, more specifically fat and muscle mass, may contribute to explaining the differences in its serum concentrations and GFR estimations. It is commonly known that creatinine as a metabolic byproduct of creatine phosphate degradation predominantly originates from skeletal muscle tissue. It is a critical biomarker for evaluating kidney function, yet it also serves as an indirect indicator of muscle mass. This correlation is of considerable importance in clinical practice, as the interpretation of serum creatinine levels necessitates an assessment of the patient's muscle mass. Consequently, individuals with reduced muscle mass exhibit decreased baseline serum creatinine concentrations while the kidneys have impaired function. In our study, muscle mass provided by BIA was positively correlated with creatinine, which remains in good agreement with the majority of previous studies [28].

Quite recently, considerable attention has been paid to the relationship between body composition measures as factors influencing CysC concentration, including elevated fat mass. The results obtained by numerous authors, including various patient populations, suggest that increased fat mass is associated with increased CysC concentration and consequently decreased GFR estimations from CysC [29–31]. This relationship is particularly pertinent in patients with renal diseases, where accurate assessment of kidney function is crucial. It is particularly important in MMC patients, where aberrations in body composition are common, but little data about associations between body composition measurements and renal parameters are available in this population. In 2017, Dangle et al. [32] performed a study on 131 children with NB where they showed that increasing BMI was associated with decreasing eGFR estimation per CysC. However, the association of CysC with fat mass has not been extensively studied using objective measures of body composition. In our study we validated the body composition measures with BIA and demonstrated that the greater the fat mass percentage provided by BIA, the higher the CysC concentration. Additionally, our data show detection of a lower GFR in MMC patients when calculated using CysC than serum creatinine in the whole group and the lowest value in children classified as overweight/obese. Consequently, the interpretation of CysC levels and GFR equation based on CysC must account for the patient's adiposity to avoid misestimation of renal impairment.

In our study, we evaluated several GFR formulas based on creatinine alone, CysC alone, or both markers. Formulas relying solely on creatinine levels, such as the original Schwartz, updated Schwartz, and Counahan–Barratt formulas, consistently overestimated the GFR in MMC patients, consistent with findings from previous studies [5, 6, 33]. Our novel approach involved utilizing BIA-derived GFR, marking the first analysis of its kind in this patient population. A limitation is that the formula used for BIA-derived GFR is proprietary and unpublished, known only to the manufacturer, with information indicating it is based on creatinine levels. However, among the five equations incorporating creatinine, this formula yielded the lowest median GFR and demonstrated good agreement with the latest CKiD-U25 formula incorporating both creatinine and CysC. This suggests that it may provide a more accurate measure of GFR in MMC children compared to traditional estimations. A significant advantage of this method is its non-invasive nature and ease of access. The incorporation of body composition measures into such equations would represent a significant advancement in GFR estimation for patient populations with MMC and associated body composition challenges.

The present study has several strengths. Our prospective study successfully identifies the inadequacies of standard adiposity measures (such as BMI) for children with MMC, highlighting the importance of using total fat mass percentage for a more accurate assessment of overweight and obesity. Additionally, we evaluated the BIA-derived body composition parameters in subgroups of patients with MMC according to the level of physical activity. Our findings show that non-ambulatory and non-functional ambulators, as well as obese patients, exhibit the highest FMR rates. This suggests that these subgroups of MMC patients may be at heightened risk for metabolic syndrome and its long-term health consequences. Identifying high FMR rates in specific subgroups highlights the need for early intervention and tailored healthcare strategies to manage the risks of metabolic syndrome in MMC patients. Furthermore, the influence of body fat mass on CysC was analysed, which is crucial for adjusting GFR equations. This insight helps in preventing the misestimation of kidney function. eGFR equations were compared that incorporate creatinine-only, CysC-only, and both variables, including recent equations espoused by CKiD-U25, which had not been previously studied in MMC patients. Since eGFR values were compared from different equations within the same patients, any bias should be uniform and non-differential. Finally, our study is the first to report that BIA-derived GFR shows promise for accurate kidney function estimation in pediatric NB. While our study provides valuable initial insights, it also lays the groundwork for further research.

The present study has limitations. First, the sample size of 40 children with MMC might be viewed as relatively small and includes patients from a single geographic region. However, it is important to recognize that MMC is a rare congenital condition, and there is a trend globally toward pregnancy termination following a prenatal diagnosis of MMC. Secondly, as the data come from a single, large, tertiary referral centre, the results are not necessarily generalizable and must be validated externally. Furthermore, we focused on the two clinically used blood test markers, creatinine and CysC, along with their estimated equations of GFR for renal function assessment. While these markers are among the most clinically useful for assessing kidney function, this narrow focus excludes other potentially relevant factors. The inclusion of additional parameters, such as those related to high detrusor pressures, recurrent urinary tract infections, and proteinuria, could provide a more comprehensive evaluation of kidney function. Although there are various recently updated guidelines on the management of patients with MMC, there is a lack of consensus on the best way to evaluate kidney function in these patients. Our primary objective was to explore the relationship between body composition parameters, including muscle mass and fat mass obtained through BIA, and the selected renal markers, creatinine, CysC and GFR estimations.

To sum up, we would like to present the main conclusions in the point-by-point manner:

1. No standard measure of adiposity and obesity exists for children with MMC. Total fat mass percentage is more sensitive than BMI in assessing overweight and obesity.
2. BIA accurately measures total body fat mass and increases obesity rates in MMC populations.
3. CysC levels are influenced by body fat mass, necessitating adjustments in GFR equations to avoid renal impairment misestimation. Further research is needed to understand the relationship between fat mass and CysC concentrations for better kidney function assessment.
4. Existing eGFR formulas show limited precision in MMC populations, highlighting the need for a validated MMC-specific GFR formula. Clinicians should be aware of the limitations and variability of creatinine, CysC, and eGFR equations for accurate interpretation in MMC patients.
5. BIA-derived GFR shows promise for accurate estimation in pediatric NB.

## Supplementary Information

Below is the link to the electronic supplementary material.

Graphical Abstract (PPTX 151 KB)

## Data Availability

The data presented in this study are available on request from the corresponding author. The data are not publicly available for ethical and privacy reasons.
