# Supplementary material for: Is body composition important in the context of renal function in pediatric neurogenic bladder?
Source: Pediatr Nephrol. 2024 Oct 17;40(5):1677–87. doi: 10.1007/s00467-024-06557-5 (PMC11946935; doi:10.1007/s00467-024-06557-5)
Supplement: Supplementary file 1 — Graphical Abstract (PPTX 151 KB) [file 467_2024_6557_MOESM1_ESM.pptx]

## Slide 1
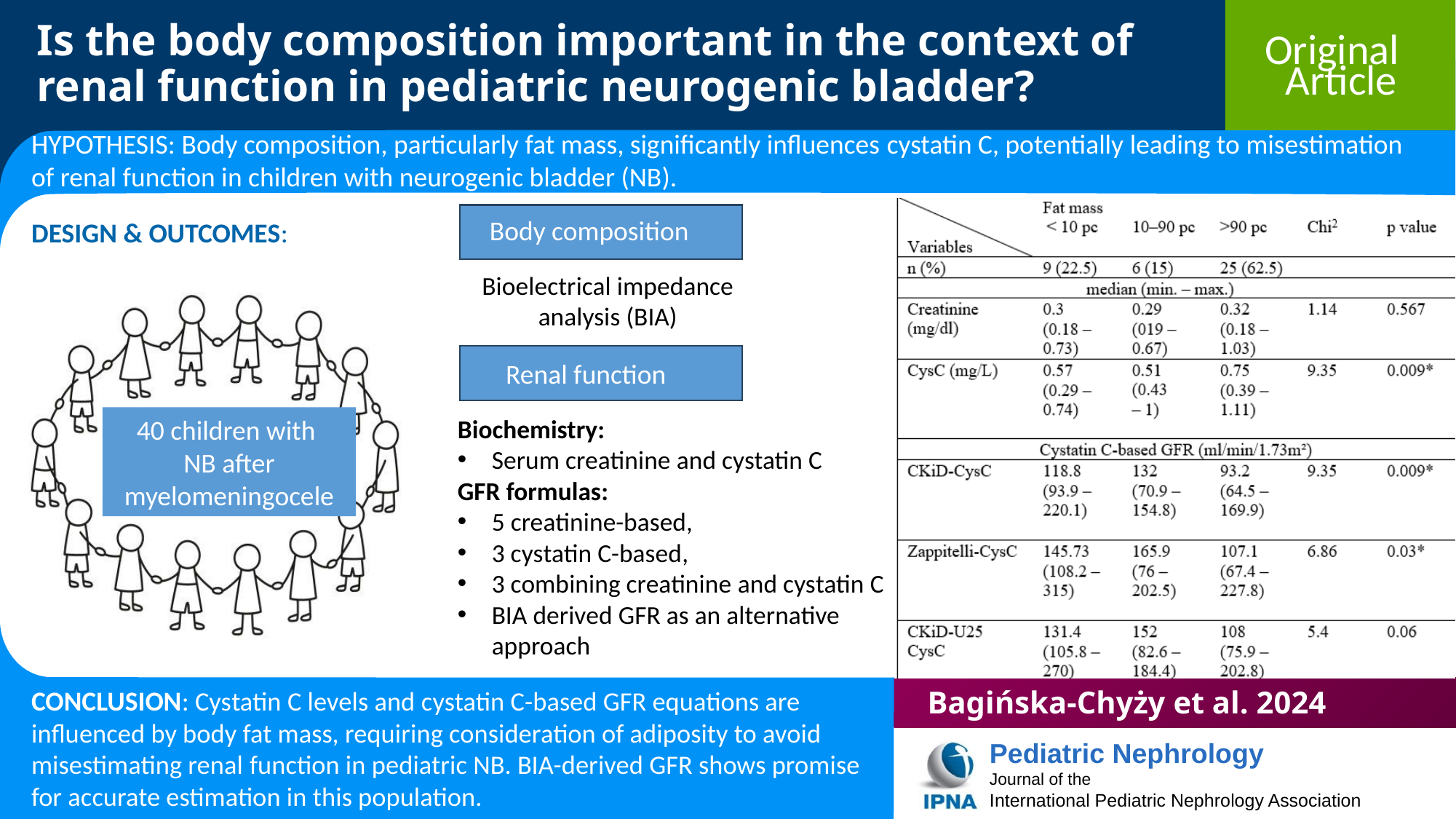

Is the body composition important in the context of renal function in pediatric neurogenic bladder?
HYPOTHESIS: Body composition, particularly fat mass, significantly influences cystatin C, potentially leading to misestimation
of renal function in children with neurogenic bladder (NB).
Body composition
DESIGN & OUTCOMES:
Bioelectrical impedance
analysis (BIA)
Renal function
40 children with
NB after myelomeningocele
Biochemistry:
Serum creatinine and cystatin C
GFR formulas:
5 creatinine-based,
3 cystatin C-based,
3 combining creatinine and cystatin C
BIA derived GFR as an alternative approach
CONCLUSION: Cystatin C levels and cystatin C-based GFR equations are influenced by body fat mass, requiring consideration of adiposity to avoid misestimating renal function in pediatric NB. BIA-derived GFR shows promise for accurate estimation in this population.
Bagińska-Chyży et al. 2024
